# Fine-Needle Aspiration Cytology of Ameloblastic Carcinoma: A Diagnostic Challenge

**DOI:** 10.7759/cureus.103316

**Published:** 2026-02-09

**Authors:** Renu Sahay, Mayank Singh, Shivanjali Singh, Sufia A Khan, Eshan Sharma

**Affiliations:** 1 Department of Pathology, Maharani Laxmi Bai Medical College, Jhansi, IND

**Keywords:** ameloblastic carcinoma, early diagnosis, fibular grafting, fine needle aspiration cytology (fnac), histopathology, keratin pearl, mandible, mandibular neoplasm, odontogenic tumours

## Abstract

Fine-needle aspiration cytology (FNAC) has been extensively applied in the evaluation of head and neck lesions, yet its role in odontogenic tumors remains underexplored. Ameloblastic carcinoma, a rare malignant odontogenic tumor, combines histologic features of ameloblastoma and carcinoma, posing a diagnostic challenge. We report the case of an 83-year-old woman who presented with a slow-growing, painless swelling over the anterior chin associated with numbness and loosening of teeth. Her medical history included partial mandibulectomy with plating six years prior following a mandibular fracture, though operative records were unavailable. FNAC of the swelling yielded mucinous to reddish-brown aspirate, with cytology revealing basaloid cells arranged in a palisading pattern along with stellate-shaped cells, initially suggestive of ameloblastoma. However, the presence of significant cellular atypia and keratin pearl formation raised suspicion of ameloblastic carcinoma. Based on these findings, a provisional diagnosis of ameloblastic carcinoma was made. The patient subsequently underwent surgical resection with fibular grafting under general anesthesia, and histopathological evaluation confirmed the diagnosis, clearly distinguishing it from its benign counterpart. This case highlights the diagnostic value of FNAC in odontogenic malignancies, demonstrating its potential as a minimally invasive, cost-effective, and reliable tool for preoperative diagnosis. Incorporating FNAC into the diagnostic algorithm for suspected odontogenic tumors may improve surgical planning and overall management, particularly in distinguishing aggressive variants such as ameloblastic carcinoma, thereby reducing recurrence and optimizing prognosis.

## Introduction

Ameloblastic carcinoma is an infrequent odontogenic epithelial malignancy that histopathologically mirrors ameloblastoma but is distinguished by pronounced cytologic atypia and aggressive biological course. The tumour accounts for under 2% of all odontogenic lesions yet represents nearly one-third of the malignant lesions [1]. Elzay in 1982 was the first to define ameloblastic carcinoma with the histological features of ameloblastoma with a malignant squamous component [2].

Epidemiology

Epidemiological data emphasize the rarity of ameloblastic carcinoma with an estimated global incidence of 0.5 per million person-years [3]. In a comprehensive 40-year scoping review that included 80, Cristofaro et al. reported its strong propensity for the posterior mandible, a male-to-female ratio of 2.03:1, and a mean age of presentation at 48.6 years; interestingly, women presented earlier, at a mean of 37.7 years, compared with men, whose mean was 53.7 years [4].

Molecular and immunohistochemical profile

Ameloblastic carcinoma often shares morphological features with ameloblastoma, but acquires molecular alterations associated with malignant transformation and aggressive behavior [5,6]. Contemporary molecular synthesis indicates that, while ameloblastic carcinoma may retain canonical odontogenic drivers seen in ameloblastoma (notably MAPK/ERK pathway activation), it more frequently demonstrates additional hits in cell-cycle and chromatin-remodeling pathways, including alterations of CDKN2A/B, TP53, RB1, and SMARCA4, supporting a model of stepwise progression from benign epithelium to carcinoma [4]. Although BRAF p.V600E has been documented in ameloblastic carcinoma, its diagnostic value for distinguishing ameloblastic carcinoma from ameloblastoma remains uncertain, reinforcing that malignant behavior likely requires cooperating events beyond MAPK activation [6].

Immunophenotypically, ameloblastic carcinoma shows a robust odontogenic-squamoid epithelial signature with pan-cytokeratin (AE1/AE3), CK5/6, and CK19 positivity, alongside basal-cell transcription factors p63 and p40 (ΔNp63) that support basal/myoepithelial-like differentiation [7,8]. Along with this, proliferation indices of Ki-67 are characteristically high, aiding distinction from ameloblastoma and correlating with aggressive biology [8,9]. Among all these discriminators, a 2024 systematic review and meta-analysis found SOX2 to be the only immunohistochemical (IHC) marker with consistent differential expression favoring ameloblastic carcinoma over ameloblastoma, highlighting its practical value in a minimal diagnostic panel, only when paired with morphology and proliferation markers [10]. After taking together, a streamlined IHC panel, p63, p40, pan-CK (AE1/AE3), CK19, and Ki-67, optionally supplemented by SOX2 and p53-integrated with clinicoradiology and awareness of recurrent molecular lesions, provides a pragmatic framework for recognizing AC and contextualizing its pathogenesis [5-10].

Role of fine-needle aspiration cytology (FNAC)

Although histopathological examination remains the gold standard for the diagnosis of odontogenic tumors, FNAC offers several practical advantages as an initial diagnostic modality. FNAC is minimally invasive, rapid, cost-effective, and can be performed on an outpatient basis, allowing early stratification of lesions into benign versus potentially malignant categories. Cytologic features that may suggest ameloblastic carcinoma include cohesive basaloid clusters with peripheral palisading, nuclear pleomorphism, prominent mitoses, necrotic background, and foci of squamoid differentiation. However, these findings may overlap with those of benign ameloblastoma, primary intraosseous squamous cell carcinoma, metastatic carcinoma to jaw, odontogenic carcinoma (NOS), and basaloid squamous cell carcinoma, which limits its diagnostic specificity [11]. The limited number of published FNAC-based diagnoses in ameloblastic carcinoma reflects diagnostic caution rather than lack of utility. FNAC has inherent limitations, including a lack of architectural assessment and cytomorphologic overlap with benign ameloblastoma. Nevertheless, cytologic features such as atypia, keratinization, squamoid differentiation, and tumor giant cells may raise early suspicion of malignant transformation and guide timely management [12]. FNAC should therefore be regarded as an adjunctive triage tool applicable across age groups, rather than a replacement for biopsy.

The present case describes an unusual instance of ameloblastic carcinoma diagnosed through FNAC, emphasizing its cytomorphologic hallmarks, diagnostic complexities, and the relevance of correlating cytology with current WHO classification standards and molecular patterns. This case emphasizes the importance of accurate cytological diagnosis in guiding further management and contributes to the limited but growing literature on the cytologic diagnosis of AC and other odontogenic malignancies.

## Case presentation

An 83-year-old female patient presented with a slowly enlarging painless swelling over the anterior mandible of six months' duration, associated with numbness and loosening of teeth, suggesting possible inferior alveolar nerve involvement. No regional lymphadenopathy was identified. The patient had undergone partial mandibulectomy with plating six years earlier for a mandibular fracture; operative and histologic records were unavailable, and its relevance to the current lesion was uncertain.

**Figure 1:**
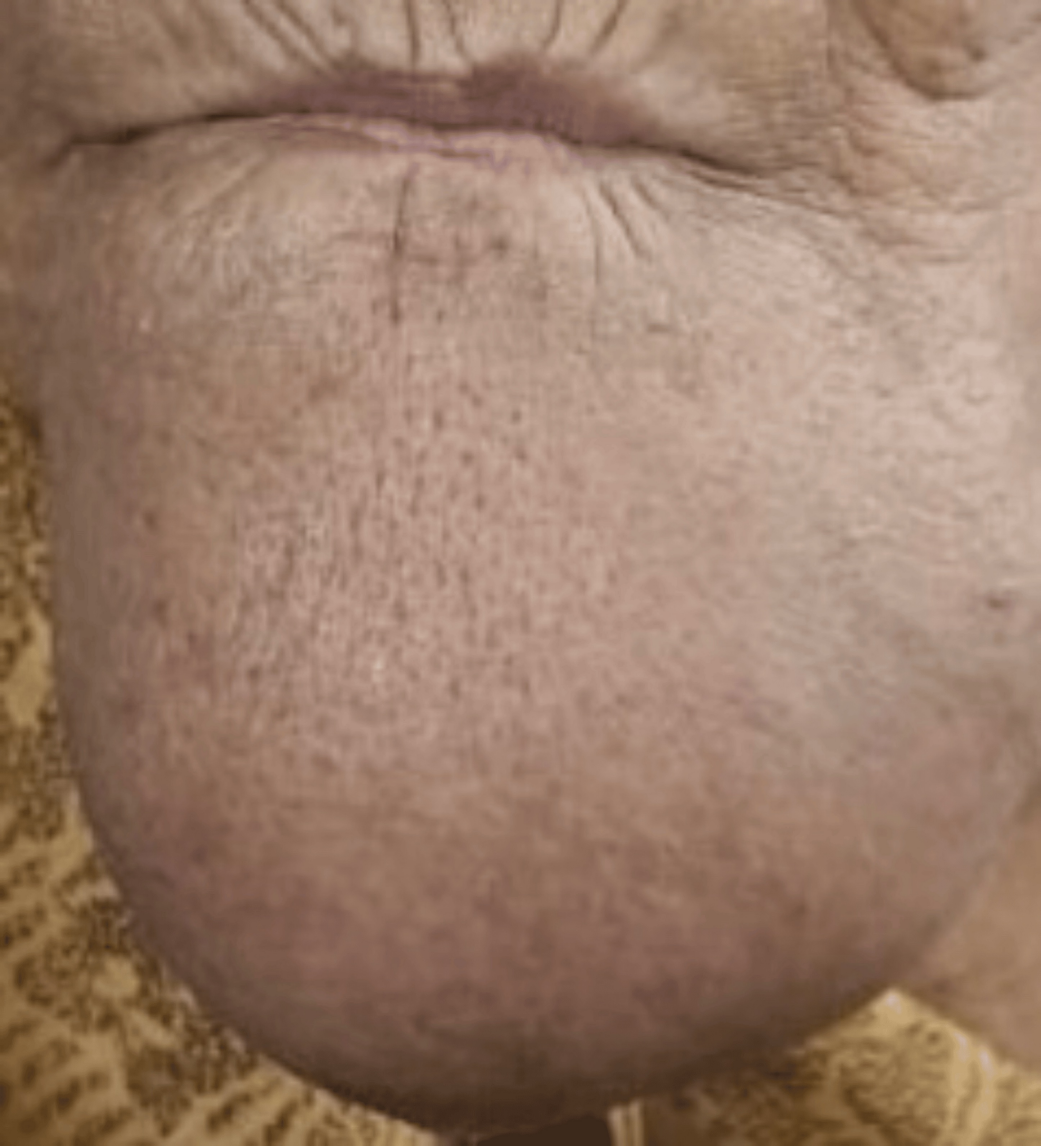
Extra-oral diffuse swelling over mental protuberance.

On clinical examination, extra-orally, a diffuse swelling was noticed on the mental protuberance (chin) (Figure 1). Palpation revealed a firm to cystic swelling measuring 8.0 × 5.0 × 5.0 cm. It was diffuse, nontender, nonreducible, nonpulsatile, noncompressible, afebrile, surface temperature and overlying skin appeared normal.

The swelling was fixed and immobile. There was no evidence of regional lymphadenopathy. Biochemical and hematological investigations were all within normal limits. General systemic examination and other relevant investigations did not reveal any metastasis.

**Figure 2:**
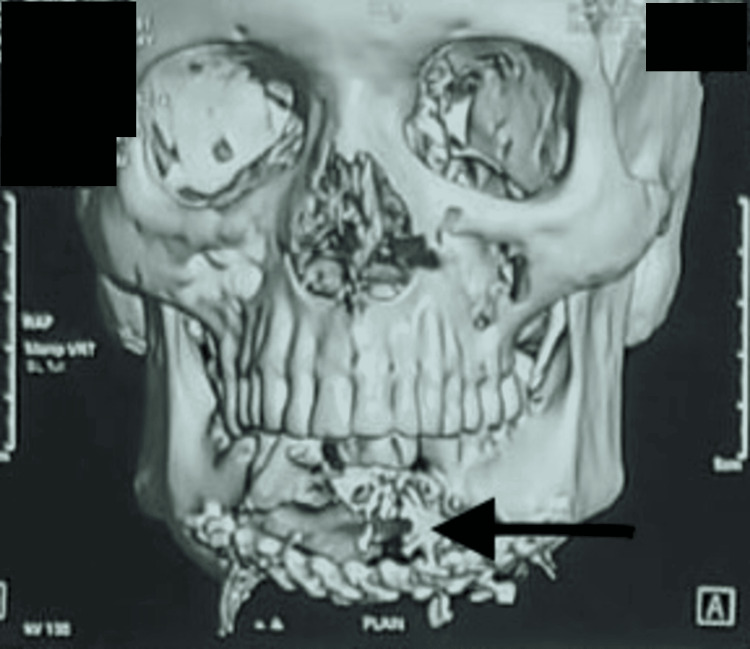
Three-dimensional reconstructed CT scan of the facial skeleton depicting an extensive, expansile, and destructive lesion involving the mandible with cortical perforation and irregular resorption (arrow).

Computed tomography (CT) revealed a multilocular, radiolucent, expansile lytic lesion involving the anterior mandible with cortical destruction. These findings are non-specific and may be seen in aggressive, benign, and malignant odontogenic tumors, though the degree of destruction suggested aggressive behavior (Figure 2).

FNAC was done by using a 22-gauge needle and a 20 cc syringe. Aspiration was done from multiple sites, and smears were fixed in 95% ethyl alcohol and air-dried. May-Grünwald Giemsa and hematoxylin and eosin staining were performed.

**Figure 3:**
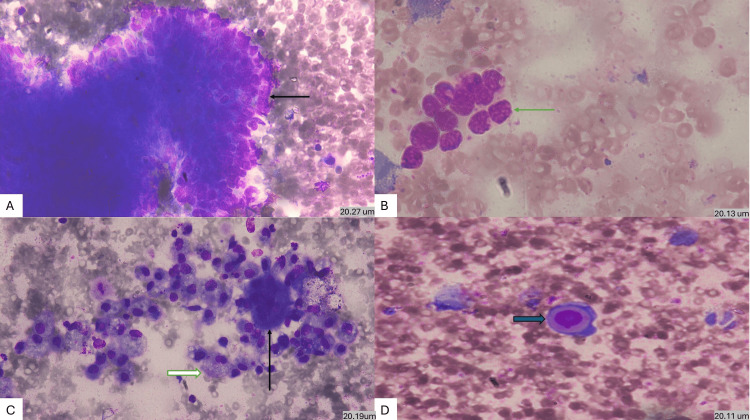
(A) MGG-stained cytology preparation showing large cohesive sheet of basaloid cells showing pseudopapillary pattern with peripheral palisading (black arrow) (20.27um) (x400). (B) Cytology preparation showing small pleomorphic cell cluster (green arrow) (20.13um) (x400). (C) Cytology preparation showing tumor giant cell (black arrow) along with cystic macrophages (white arrow) (20.19 um) (x400). (D) Cytology preparation showing intracytoplasmic keratinization (blue arrow) (20.11 um) (x400). MGG: May-Grünwald-Giemsa

Microscopically, FNAC showed cohesive sheets and nests of basaloid cells with peripheral palisading, admixed with squamoid cells, keratinous material, tumor giant cells, and moderate cytologic atypia (Figure 3). The coexistence of odontogenic morphology with cytologic malignancy raised a strong suspicion of ameloblastic carcinoma and guided definitive surgical management.

Under general anesthesia with aseptic precautions, a complete excision of the tumor mass was performed. Along with it, reconstructive surgery was done using a free fibular graft. The specimen was preserved and sent for histopathological examination.

**Figure 4:**
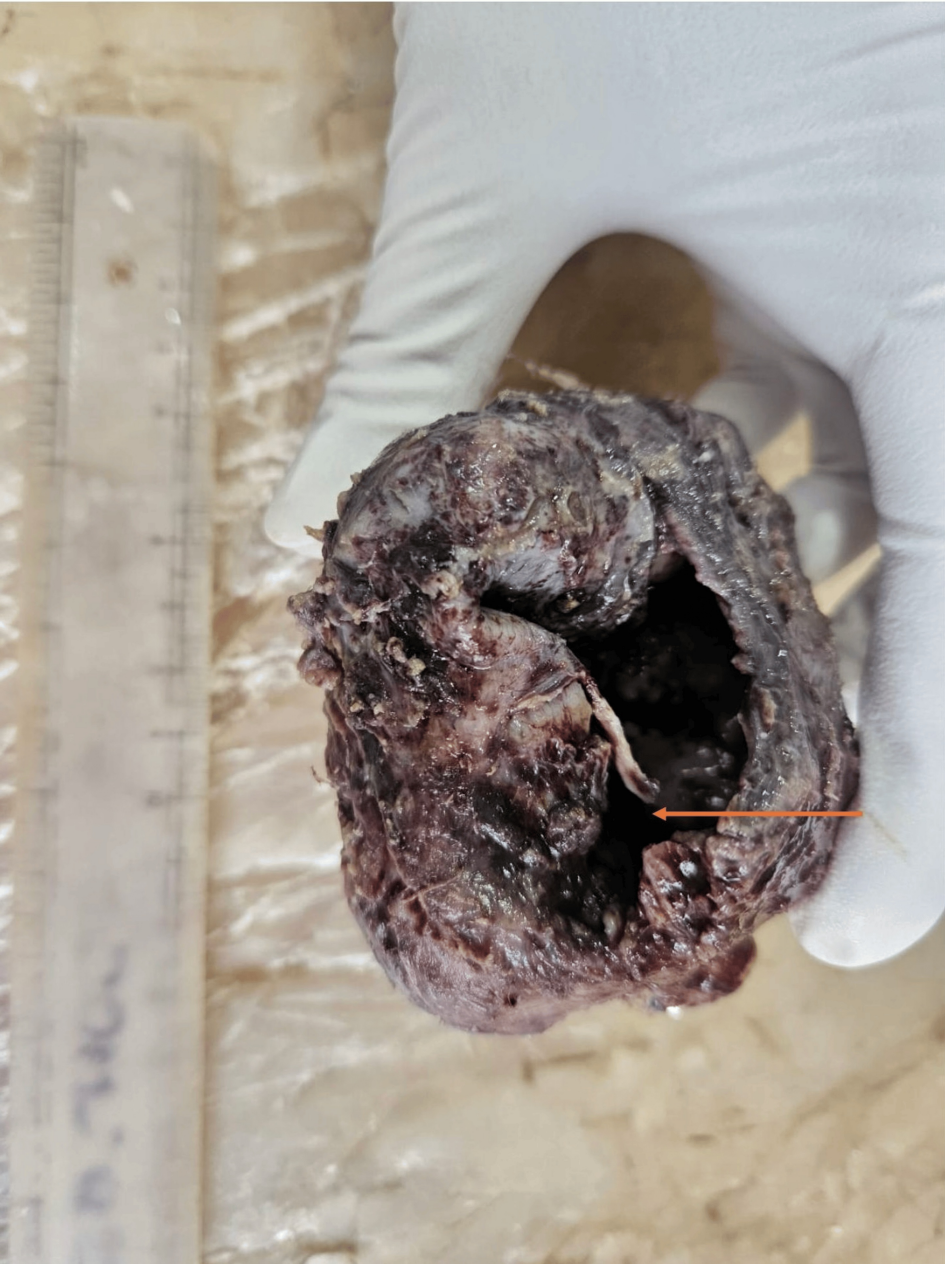
Gross image of tumor mass with large ruptured cyst (arrow).

The tumor mass measured 8.5 × 5.5 × 4.5 cm, firm to hard in consistency, with an irregular external surface (Figure 4). The cut surface showed cystic areas and solid areas with necrosis, along with a stainless steel plate, which had been used to support the chin in past mandibular surgery after the majority of the middle mandible was destroyed.

**Figure 5:**
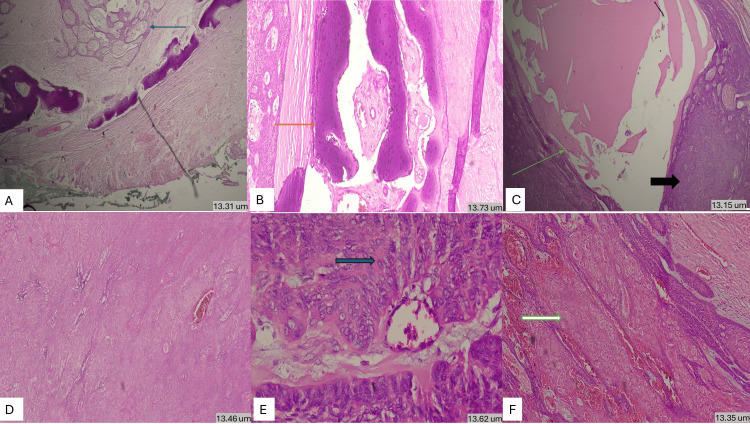
(A) H&E stained section showing tumor area composed of odontogenic palisading columnar cells arranged in plexiform pattern (blue arrow) along with uninvolved peripheral mucosa (13.31 um) (x40). (B) H&E stained section showing entrapped bony trabeculae (orange arrow) (13.73 um) (x100). (C) Section showing cystic area filled with eosinophilic mucoid material (green arrow) along with solid tumor sheet of atypical cells (black arrow) (13.15 um) (x100). (D) Section showing solid sheets of pleomorphic cells (13.46 um) (x100). (E) Section showing highly pleomorphic cells (green arrow) surrounding a blood vessel with altered nuclear-cytoplasmic ratio, hyperchromasia and prominent nucleoli along with mitotis and intervening myxoid material (13.62um) (x400). (F) Section showing tumor composed of ameloblastoma-like epithelium arranged in plexiform pattern with reversal of nuclear polarity along with islands and sheets of atypical squamous cells with intracytoplasmic keratinization (white arrow) (13.35 um) (x100). H&E: hematoxylin and eosin

Microscopically, sections showed ameloblastoma-like epithelium arranged in a plexiform pattern, composed of peripheral columnar cells with palisading and reverse polarity, and central stellate reticulum-like cells (Figure 5). Intermixed with atypical odontogenic cells, malignant squamous epithelial cells arranged in sheets, cords, and nests, with nuclear pleomorphism, hyperchromasia, prominent nucleoli, mitotic activity, and keratinization were noted. Areas of necrosis, hemorrhage, foamy macrophages, mixed inflammatory infiltrate, cystic spaces filled with eosinophilic material, and entrapped bony trabeculae were also present. Mucosa and muscle fibers were unremarkable.

**Figure 6:**
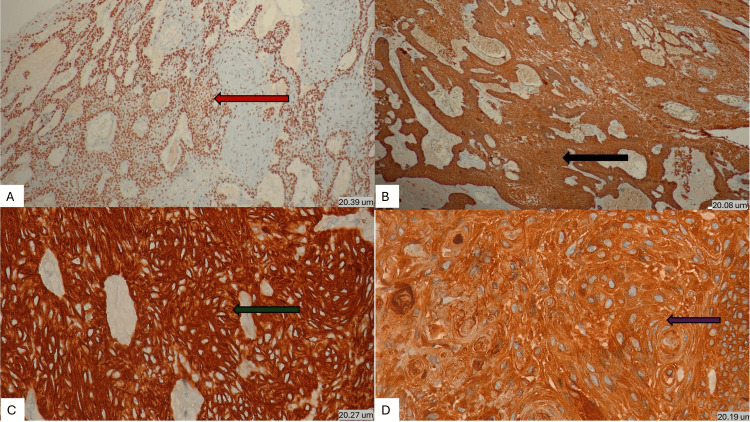
(A) IHC staining showing diffuse positivity for p40 (red arrow) (20.39 um) (x100). (B) Diffuse positivity for p63 (black arrow) (20.06 um) (IHC, x100). (C) Strong positivity for pan-cytokeratin (green arrow) (20.27 um) (IHC, x400). (D) Diffuse positivity for pan-cytokeratin (brown arrow) (20.19 um) (IHC, x1000).

Also, we performed IHC using p40, p63, Pan-CK, CK7 and BCL2 (Figure 6). The former three were diffusely positive, and the latter two were negative, overall supporting the epithelial origin of the tumor.

The postoperative period was uneventful. The patient was maintained on antibiotics and analgesics and monitored for graft viability. The free fibular graft showed good perfusion, and there were no signs of flap necrosis or infection. Oral intake was reintroduced gradually, and mandibular function improved over the next few weeks.

The patient was discharged on postoperative day 15 with instructions for regular follow-up. At the six-week follow-up, the surgical site demonstrated satisfactory healing with no evidence of recurrence clinically or radiologically. The patient reported improvement in numbness but persistent mild paraesthesia. A three-month follow-up CT scan showed stable reconstruction with no new lesions. Long-term oncologic surveillance was advised due to the known recurrence potential of ameloblastic carcinoma.

## Discussion

Our patient, an 83-year-old female, presented with a slow-growing, painless mandibular swelling associated with numbness and loosening of teeth. The lesion showed both solid and cystic areas, consistent with the aggressive biology reported in previous cases [13,14]. Radiological features demonstrated expansile destruction, and histopathology revealed basaloid nests with peripheral palisading, squamoid differentiation, keratin pearls, and atypia, findings typical of ameloblastic carcinoma [15,16].

**Table 1:** Comparison table of utility of FNAC with conventional biopsy in odontogenic tumors. FNAC: fine-needle aspiration cytology

Parameters	FNAC	Conventional Biopsy
Invasiveness	Minimal	Invasive
Turnaround time	Rapid	Longer
Architectural assessment	Limited	Complete
Cytological atypia	Well assessed	Well assessed
Malignancy suspicion	Early	Definitive
Role	Triage and suspicion	Confirmation

This case demonstrates the value of FNAC in raising early suspicion of ameloblastic carcinoma. Although FNAC cannot replace histopathology, it can identify malignant cytologic features that prompt timely intervention. Ki67 was not included in the IHC panel due to its unavailability at that time; however, definitive diagnosis was established by histopathology, emphasizing the primacy of morphology with immunohistochemistry as a supportive tool.

Collectively, IHC supports the epithelial nature of ameloblastic carcinoma and may indicate proliferation or anti-apoptotic tendencies (Ki-67, p53, BCL-2), but cannot reliably distinguish ameloblastoma from ameloblastic carcinoma. Our case emphasizes that FNAC may provide earlier clues to malignant transformation than IHC, reinforcing its value as a key early diagnostic and prognostic tool.

Treatment of ameloblastic carcinoma primarily involves wide resection with clear margins. Reports have consistently shown aggressive surgery to be the gold standard [14,17], with radiotherapy reserved for recurrences or incomplete excision [18]. Prognosis is determined mainly by local aggressiveness rather than distant spread, though long-term surveillance is required.

This case highlights that FNAC combined with histology provides the strongest diagnostic pathway, while IHC remains a supportive adjunct rather than a defining criterion.

## Conclusions

Ameloblastic carcinoma is a rare but aggressive odontogenic malignancy that requires a high index of suspicion for early diagnosis. The clinical overlap with benign ameloblastoma often delays recognition, underscoring the importance of integrating radiological, cytological, and histopathological findings.

In our case, FNAC played a crucial role by demonstrating basaloid cell clusters with keratinization and moderate atypia, which strongly favored a suspicion of malignant odontogenic tumor. Early and accurate identification remains essential to guide surgical management. FNAC is a valuable adjunctive diagnostic tool in the early assessment of aggressive odontogenic tumors, while histopathology remains essential for definitive diagnosis.

## References

[1] (2024). Head and Neck Tumours. WHO Classification of Tumours, Vol 9. http://publications.iarc.who.int/Book-And-Report-Series/Who-Classification-Of-Tumours/Head-And-Neck-Tumours-2024.

[2] Elzay RP (1982). Primary intraosseous carcinoma of the jaws: review and update of odontogenic carcinomas. Oral Surg Oral Med Oral Pathol.

[3] Reza N, Qader OA, AL-Rawas M, Omar M, Abdullah JY, Satmi AS (2025). Ameloblastic carcinoma of the jaws: a comprehensive review of current perspectives and emerging trends. Glob J Med Pharma Biomed Update.

[4] Cristofaro MG, Barca I, Sottile AR, Ferragina F (2025). Ameloblastic carcinoma: a 40-year scoping review of the literature. Curr Issues Mol Biol.

[5] Garg N, Krishna R, Urs AB, Kumar P, Augustine J (2025). A systematic review on the molecular pathways of ameloblastic carcinoma when compared to ameloblastoma. Expert Rev Mol Diagn.

[6] Tomar S, Tomar U, Singh R, Verma N (2023). An interesting case-report of ex-ameloblastic carcinoma. J Oral Maxillofac Pathol.

[7] Chen YF, Hsu PK, Shen CR (2023). Maxillary ameloblastic carcinoma mimicking conventional ameloblastoma: clinicopathological, immunohistochemical, and molecular genetic study. Int J Surg Pathol.

[8] McNaught MJ, Turella SJ, Fallah DM, Demsar WJ (2015). Spindle cell variant of ameloblastic carcinoma: a case report and review of literature. Mil Med.

[9] Martínez-Martínez M, Mosqueda-Taylor A, Carlos-Bregni R (2017). Comparative histological and immunohistochemical study of ameloblastomas and ameloblastic carcinomas. Med Oral Patol Oral Cir Bucal.

[10] Mishra S, Panda S, Mohanty N, Mishra S, Gopinath D, Panda S, Anil S (2024). Differential expression of immunohistochemical markers in ameloblastoma and ameloblastic carcinoma: a systematic review and meta-analysis of observational studies. F1000Res.

[11] Nai GA, de Almeida Freitas R, de Oliveira MG, Santos PS, de Oliveira RB (2011). Fine-needle aspiration biopsy of ameloblastic carcinoma of the mandible: a case report. Braz Dent J.

[12] Ingram EA, Evans ML, Zitsch RP 3rd (1996). Fine-needle aspiration cytology of ameloblastic carcinoma of the maxilla: a rare tumor. Diagn Cytopathol.

[13] Dhir K, Sciubba JJ, Tufano RP (2003). Ameloblastic carcinoma of the maxilla. Oral Oncol.

[14] Kruse AL, Zwahlen RA, Grätz KW (2009). New classification of maxillary ameloblastic carcinoma based on an evidence-based literature review over the last 60 years. Head Neck Oncol.

[15] Canales NA, Marina VM, Castro JS (2014). A1BG and C3 are overexpressed in patients with cervical intraepithelial neoplasia III. Oncol Lett.

[16] Casaroto AN, Toledo GL, Filho FJ, Soares CT, Capelari MM, Lara VS (2012). Ameloblastic carcinoma, primary type: case report, immunohistochemical analysis and literature review. Anticancer Res.

[17] Soyele OO, Adebiyi KE, Adesina OM, Ladeji AM, Aborisade A, Olatunji A, Adeola HA (2018). Ameloblastic carcinoma: a clinicopathologic analysis of cases seen in a Nigerian teaching hospital and review of literature. Pan Afr Med J.

[18] Aoki T, Akiba T, Kondo Y, Sasaki M, Kajiwara H, Ota Y (2019). The use of radiation therapy in the definitive management of ameloblastic carcinoma: a case report. Oral Surg Oral Med Oral Pathol Oral Radiol.

